# KH906, a recombinant human VEGF receptor fusion protein, is a new effective topical treatment for corneal neovascularization

**Published:** 2011-03-25

**Authors:** Tao Li, Andina Hu, Shiqing Li, Yan Luo, Juan Huang, Honghua Yu, Wei Ma, Jianying Pan, Qi Zhong, Jin Yang, Jianming Wu, Shibo Tang

**Affiliations:** 1 State Key Laboratory of Ophthalmology, Zhongshan Ophthalmic Center of Sun Yat-sen University, Guangzhou 510060, China; 2 Chengdu Kanghong Biotechnology Co. Ltd, Chengdu, China

## Abstract

**Purpose:**

The purpose of the current study was to investigate the effect of topical administration of KH906 on corneal neovascularization (NV).

**Methods:**

To induce corneal neovascularization, chemical cauterization of the corneas of the right eyes of forty-eight New Zealand white rabbits was performed by touching the central cornea with an 8-mm-diameter NaOH-soaked Whatman filter paper for 60 s. On the next day after modeling, the rabbits were randomly and equally divided into six groups: PBS control group, 0.1% dexamethasone group, 10 mg/ml Avastin group, 5 mg/ml KH906 group, 10 mg/ml KH906 group, and 20 mg/ml KH906 group. The rabbits in the six groups received topical administration of 50 μl of the different solutions on the cornea four times per day for 14 days. Corneal neovascularization was analyzed by slit-lamp biomicroscopy 10 and 14 days after chemical cauterization. Corneal fluorescein staining was performed to evaluate the extent of corneal epithelial defect on the 7th, 10th, and 14th days. The VEGF level of the cornea was evaluated by ELISA assay.

**Results:**

On the 10th and 14th days after chemical cauterization, the length of the longest new vessel and the areas of corneal neovascularization in all KH906-treated groups were significantly reduced compared to those of the PBS-treated group (p<0.05). The VEGF level of the cornea in all KH906-treated groups was significantly decreased compared to that of the PBS-treated group (p<0.05). Corneal fluorescein staining showed that KH906 had no effect on corneal epithelial healing.

**Conclusions:**

Topical administration of KH906 significantly inhibited alkali burn-induced corneal neovascularization in rabbits. The new eye drops of KH906 may have a broad application for human corneal neovascularization in the near future.

## Introduction

In normal physiologic status, the cornea is transparent to maintain normal vision. Corneal neovascularization is a concomitant complication of many ocular surface diseases. It significantly diminishes corneal clarity and is a cause of a subsequent reduction in vision [1]. Meanwhile, corneal neovascularization is the major reason for corneal allograft rejection [2,3]. Various treatments including drugs and surgery have been applied in treating corneal neovascularization. Surgical operations such as radiotherapy, laser therapy, photodynamic therapy, and diathermy are very complicated. Drugs including steroids, methotrexate, heparin, cyclosporine A, and thalidomide are used but do not currently produce an ideal clinical effect [1,4-7]. Recently, vascular endothelial growth factor (VEGF) has been proven a major inducer of corneal neovascularization [7-9]. VEGF and its tyrosine kinase receptors (flt-1 and KDR) are key mediators in angiogenesis.

Corneal neovascularization occurs as a result of a disequilibrium between angiogenic and antiangiogenic factors [4]. Anti-VEGF treatment has shown great results in many animal models and clinical trials [10,11]. Avastin (Bevacizumab; Genentech, San Francisco, CA) is a typical anti-angiogenesis medicine that has entered the clinical trial stage to treat corneal neovascularization. This drug is a recombinant humanized monoclonal IgG1 antibody with a 93% human and 7% murine protein sequence. Avastin binds to all five VEGF-A isoforms. It is already being administered intravitreally for the treatment of exudative age-related macular degeneration, proliferative diabetic retinopathy, and iris rubeosis with good results [12-15]. Subconjunctival administration of Avastin in experimental animal models could inhibit corneal neovascularization [16-18].

KH906, a recombinant human soluble endothelial growth factor (VEGF) receptor fusion protein with a 100% human protein sequence, is the patented product of Chengdu Kanghong Biotechnology Co. Ltd. (Chengdu, China). KH906 contains extracellular ligand-binding domain 3, 4 from VEGFR2/KDR, domain 2 from VEGFR1/ Flt-1, and human IgG Fc protein. A VEGF binding assay and a VEGF-induced HUVECs proliferation assay demonstrated that KH906 could bind VEGF with a very high affinity and inhibit VEGF-induced HUVECs proliferation. KH906 binds to not only all VEGF-A isoforms, but also VEGF-B and VEGF-C, so it has a stronger anti-angiogenesis effect than Avastin in theory. KH902 (Chengdu Kanghong Biotechnology Co. Ltd., Chengdu, China), another molecule with the same amino acid sequence but different production processes and glycosylation, was proven effective in animal models for treating choroidal neovascularization (CNV) [19]. Therefore, this study was designed to investigate the effect of the topical administration of KH906 on the prevention of alkali burn-induced corneal neovascularization in a rabbit model.

## Methods

### Animals

This study was approved by the Institutional Animal Care and Use Committee of Zhongshan Ophthalmic Center, Sun Yat-sen University, Guangzhou, China. All experimental procedures on animals were conducted in accordance with the Association for Research in Vision and Ophthalmology Resolution on the use of animals in research. Forty-eight New Zealand white rabbits weighing between 2.2 kg and 2.5 kg were used in the study. The animals were housed under a 12 h:12 h light-dark cycle with standard chow and water ad libitum.

### Induction of corneal neovascularization

General anesthesia was induced by an intramuscular injection of ketamine HCl (2 mg/kg bodyweight) and chlorpromazine HCl (2 mg/kg bodyweight) [20]. At the same time, more than 50 round filter papers (8-mm diameter) were prepared and immersed in 10 μl 1M NaOH for 10 s. While the animals were under general anesthesia, the NaOH-soaked filter paper was placed on the center of the cornea surface of the right eyes of the rabbits for 60 s. Then, the eyes were rinsed thoroughly with more than 50 ml sterile normal saline. To prevent potential infection, all burned eyes were treated with erythromycin eye ointment. To increase the reproducibility of the alkali burn, the whole process was performed on each rabbit by the same investigator.

### Groups and topical administration of solutions on corneas

On the next day after modeling, the rabbits were randomly divided into six groups: PBS control group, 0.1% dexamethasone group, 10 mg/ml Avastin group, 5 mg/ml KH906 group, 10 mg/ml KH906 group, and 20 mg/ml KH906 group. The rabbits in the six groups received topical administration of 50 μl of different solutions on the cornea four times per day for 14 days.

### Observation and examination

The chemical cauterization modeling day was taken as day 0. All eyes were examined daily, and major reports on the development of corneal neovascularization were made 10 and 14 days after chemical cauterization by slit-lamp examination (SL-120; Zeiss, Jena, Germany). To minimize observer bias, all observations were performed by an eye doctor who was blinded to the allocation of the animals in each group. Digital photographs were obtained with a Nikon megapixels digital camera (D90; Nikon imaging [China] Sales Co. Ltd, Beijing, China). The area of corneal neovascularization was determined using the following formula: AC=C/12×3.1416×[r^2^- (r-L) ^2^] [21,22] where C represents the clock hours of corneal neovascularization, r represents the radius of the animal cornea, and L represents the length of the longest new vessel. The length of vessel was measured with a reticule from the limbus to the tip of the vessel. All photographs were evaluated by two investigators.

On day 15 after chemical cauterization, all animals were sacrificed, and the corneas were harvested. The homogenate of each cornea was taken for the ELISA assay to examine VEGF levels with a fully automatic enzyme-linked analyzer.

In addition, corneal fluorescein staining with fluorescein sodium was performed on the 7th, 10th, and 14th days to evaluate corneal epithelial healing. The area of epithelial defect was measured using Image-Pro Plus 6.0 software.

### Statistical analysis

All data in each group was reported as the mean±SD. The significant differences between the treated and control groups were determined using the one-way ANOVA. Statistical analysis was performed using SPSS software version 16.0 for Windows (SPSS Inc., Chicago, IL). A value of p<0.05 was considered significant.

## Results

### Corneal neovascularization after alkali burn

**Figure 1:**
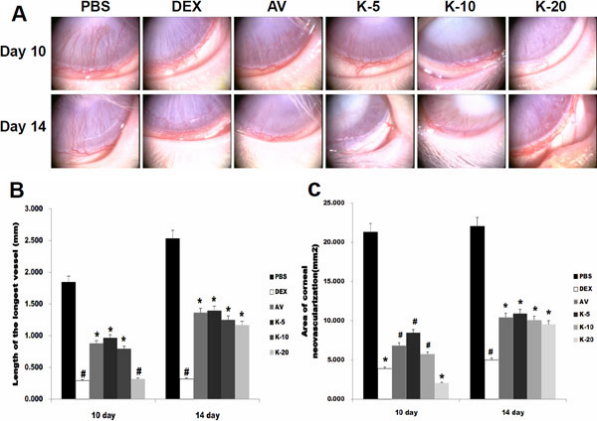
Corneal NV induced by alkali burn. **A**: slit-lamp photograph of corneal NV on the 10th and 14th days after chemical cauterization. The NV of the PBS-treated group was dense, fine, and almost reached to the central cornea. The NV of all KH906-treated groups and the Avastin-treated group was less dense and shorter than that of the PBS group. The inhibitive effect on NV in the dexamethasone-treated group was the best. **B**: The length of the longest vessel in all groups on the 10th and 14th days. The mean length of the longest new vessel in all medicine-treated groups was shorter than that of the PBS control group on both the 10th and 14th days after cauterization (p<0.05). The vessel length was shortest in both the dexamethasone-treated group and the KH906 (20 mg/ml)-treated group, and there was a statistically significant difference between the two groups and the other groups on the 10th day (p<0.05). Meanwhile, only the length of the longest vessel in the dexamethasone-treated group differed significantly from those of the other medicine-treated groups on the 14th day (p<0.05). **C**: Area of corneal NV in all groups on the 10th and 14th days. The change in the area of corneal NV showed the same trend as that of the length of the longest new vessel. PBS: PBS, DEX: dexamethasone, AV: Avastin, K-5:KH906 (5 mg/ml), K-10:KH906 (10 mg/ml), K-20:KH906 (20 mg/ml). *There is a statistically significant difference between groups with a *symbol and groups without a *symbol. #There is a statistically significant difference between groups with a #symbol and groups without a #symbol.

During the entire experimental period, no endophthalmitis or inflammation of the ocular surface was observed. In the corneal alkali burn model, neovascular began to form within the first week, and mild peripheral neovascularization was apparent on the 7th day. The vessels that emerged from the adjacent limbus appeared short, fine, and dense. Two weeks after cauterization, there was clear evidence of neovascularization with major visible vessels. The corneal neovascularization of the PBS control group (PBS), 0.1% dexamethasone group (DEX), 10 mg/ml Avastin group (AV), 5 mg/ml KH906 group (K-5), 10 mg/ml KH906 group (K-10), and 20 mg/ml KH906 group (K-20) on the 10th and 14th days are shown in Figure 1A.

On the 10th day after cauterization, the length of the longest new vessel showed a significant decrease in all medicine-treated groups compared with the PBS control group (AV=0.88±0.57 mm, K-5=0.97±0.52 mm, K-10=0.80±0.84 mm *p<0.05 versus PBS=1.85±0.67 mm ; DEX=0.29±0.09 mm, K-20=0.32±0.33 mm #p<0.05 versus PBS). The 0.1% dexamethasone-treated group and the 20 mg/ml KH906-treated group showed greater results than the other medicine-treated groups. On the 14th day after cauterization, the length of the longest new vessel also showed a significant decrease in all medicine-treated groups compared with the PBS control group (AV=1.36±1.40 mm, K-5=1.40±1.38 mm, K-10=1.25±0.94 mm, K-20=1.17±0.61 mm *p<0.05 versus PBS=2.53±1.23 mm; DEX=0.32±0.18 mm, #p<0.05 versus PBS). The 0.1% dexamethasone-treated group showed greater results than the other medicine-treated groups (Figure 1B).

On the 10 th day after cauterization, the area of vascularized cornea showed a significant decrease in all medicine-treated groups compared with the PBS control group (AV=6.85±3.96 mm^2^, K-5=8.47±3.58 mm^2^, K-10=5.75±3.70 mm^2^ *p<0.05 versus PBS=21.31±4.51 mm^2^; DEX=3.91±2.75 mm^2^, K-20=2.07±2.32 mm^2^ #p<0.05 versus PBS). The 0.1% dexamethasone-treated group and the 20 mg/ml KH906-treated group showed greater results than the other medicine-treated groups. On the 14th day after cauterization, the area of vascularized cornea also showed a significant decrease in all medicine-treated groups compared with the PBS control group (AV=10.45±4.50 mm^2^, K-5=10.92±6.58 mm^2^, K-10=10.08±5.13 mm^2^, K-20=9.53±4.22 mm^2^ *p<0.05 versus PBS=22.06±7.61 mm^2^ ; DEX=5.00±3.13 mm^2^, #p<0.05 versus PBS). The 0.1% dexamethasone-treated group showed greater results than the other medicine-treated groups (Figure 1C).

### VEGF ELISA assay

**Figure 2:**
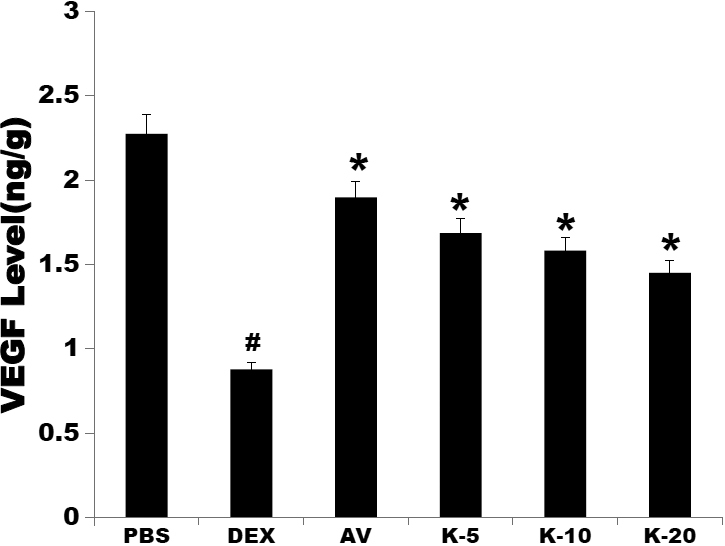
VEGF levels in the cornea. The results showed that the VEGF levels of all medicine-treated groups obviously decreased compared to that of the PBS group (p<0.05). The VEGF level was lowest in the dexamethasone-treated group, and there was a statistically significant difference between the dexamethasone-treated group and the other groups (p<0.05). All doses of KH906 decreased VEGF levels. There was no statistically significant difference between the Avastin-treated group and the KH906-treated groups (p>0.05). PBS: PBS, DEX: dexamethasone, AV: Avastin, K-5:KH906 (5 mg/ml), K-10:KH906 (10 mg/ml), K-20:KH906 (20 mg/ml).

VEGF levels in the homogenate of the cornea showed a significant decrease in all medicine-treated groups compared with those of the PBS control group (AV=1.90±0.40 ng/g, K-5=1.69±0.62 ng/g, K-10=1.58±0.26 ng/g, K-20=1.45±0.64 ng/g *p<0.05 versus PBS=2.28±0.29 ng/g; DEX=0.88±0.30 ng/g #p<0.05 versus PBS). The VEGF level was lowest in the dexamethasone-treated group. There was no statistically significant difference between the Avastin-treated group and the KH906-treated groups (p>0.05; Figure 2).

### Corneal fluorescein staining

**Figure 3:**
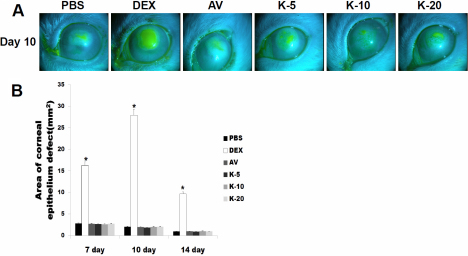
Corneal fluorescein staining on the 7th, 10th, and 14th days after chemical cauterization. **A**: Slit-lamp photograph of corneal fluorescein staining on day 10. The recovery of the corneal epithelium was strongly delayed in the dexamethasone-treated group. **B**: A statistical comparison of the area of corneal epithelial defect on the 7th, 10th, and 14th days after chemical cauterization. There was a statistically significant difference between the dexamethasone-treated group and the other groups. PBS: PBS, DEX: dexamethasone, AV: Avastin, K-5:KH906 (5 mg/ml), K-10:KH906 (10 mg/ml), K-20:KH906 (20 mg/ml).

Corneal fluorescein staining showed the effect of topical use of KH906 and other medicines on corneal wound healing after alkali burn. Results indicated that corneal damage can be repaired by the self-repair function of the cornea after alkali burn. The PBS group and all medicine-treated groups except for the dexamethasone-treated group showed fine healing processes after alkali burn (Figure 3A).

On the 7th day after cauterization, the area of epithelial defect was larger in the dexamethasone-treated group than in the other groups (PBS=2.82±0.27 mm^2^, AV=2.7±0.11 mm^2^, K-5=2.63±0.35 mm^2^, K-10=2.61±0.37 mm^2^, K-20=2.74±0.66 mm^2^ *p<0.05 versus DEX=16.27±1.78 mm^2^). On the 10th day after cauterization, there was a statistically significant difference between the dexamethasone-treated group and the other groups (PBS=2.02±0.16 mm^2^, AV=1.97±0.06 mm^2^, K-5=1.84±0.44 mm^2^, K-10=1.98±0.27 mm^2^, K-20=2.01±0.36 mm^2^ *p<0.05 versus DEX=27.92.91±2.40 mm^2^). The same trend (PBS=0.94±0.08 mm^2^, AV=0.99±0.23 mm^2^, K-5=0.92±0.17 mm^2^, K-10=0.99±0.24 mm^2^, K-20=0.96±0.08 mm^2^ *p<0.05 versus DEX=27.92±2.40 mm^2^) was apparent on the 14th day after cauterization (Figure 3B).

## Discussion

Many ocular surface diseases, such as inflammatory, infectious, degenerative, and traumatic disorders, will induce corneal neovascularization [23]. Although corneal neovascularization may play a certain role in infection removal and wound healing, it can destroy the normal corneal microenvironment and lead to the loss of the immune privilege of the cornea. The direct negative result of corneal neovascularization is the loss of corneal transparency, which is critical for vision. What is more, serious corneal neovascularization can lead to blindness.

Many drugs and operations are presently being used to treat corneal neovascularization. Steroids remain the first choice in clinical treatment on corneal neovascularization, because neovascularization is assumed to be secondary to some degree of inflammation. However, steroids are not ideal in treating corneal neovascularization, as they can cause the replication of pathogens such as fungus and herpes simplex virus and retard corneal wound healing [24-26]. Besides, steroid treatment may cause glaucoma and cataract. These side effects limit the application of steroids in corneal neovascularization. When inflammation is not the main cause of angiogenesis, such as in diseases associated with deficiency of limbal cells or corneal hypoxia, anti-inflammatory corticosteroids have little or no effect on capillary growth [16]. Laser photocoagulation, diathermy, and photodynamic therapy are also effective in the obliteration of local corneal neovascularization [27-30]. Although favorable results have been obtained with these techniques, they are not available for extensive corneal neovascularization and have complicated manipulations [31]. For all the above reasons, more effective, easier and safer corneal neovascularization treatment is required.

Recently, researchers found that VEGF acts as the key mediator during inflammation and neovascularization. It regulates the growth of vascular endothelium and controls the formation of new blood vessels [32]. VEGF is secreted mainly from macrophages, T cells, retinal pigment epithelial cells, smooth muscle cells, and tumor cells with the stimulation of various environmental factors, especially hypoxia. Binding to its receptors, VEGF triggers a signaling cascade that promotes endothelial cell growth, survival, migration, differentiation, and mobilization of endothelial progenitor cells from the bone marrow into the peripheral circulation [33]. Several anti-VEGF drugs have been tested as treatments for angiogenesis diseases. Avastin, Lucentis, Macugen, and VEGF-Trap have been applied in treating cancer, neovascular age-related macular degeneration, diabetic retinopathy, neovascular glaucoma, and corneal neovascularization. Avastin is considered one of the most effective medicines. In this study, we compared the effects of topical administration of KH906 with those of Avastin on treating corneal neovascularization.

Our results proved that both KH906 and Avastin could inhibit corneal neovascularization equally with the same decrease of VEGF level in cornea. At the same dose of tropic administration on cornea, there was no statistically significant difference on inhibiting the corneal neovascularization between KH906-treated group and Avastin-treated group. KH906 was proved very effective in preventing neovascularization when administered topically. The length and area of corneal neovascularization decreased significantly compared with the PBS control group. Although KH906 seemed less effective than dexamethasone, its safety makes it a promising drug. In our study, corneal fluorescein staining showed that the topical use of KH906 had no side effects on the processes of corneal wound healing, but dexamethasone seriously influenced corneal wound healing. As we discussed above, the side effects limit the applications of steroids in corneal neovascularization. KH906 is a fully humanized protein that can be absorbed without obvious side effects.

To conclude, topical administration of KH906 is effective in treating corneal neovascularization in the rabbit experimental model. No adverse effects on the cornea were found. More research is needed to define the ideal concentration and time of administration of KH906 to achieve the best clinical outcome. The new eye drops of KH906 may have a broad application in the treatment of human corneal neovascularization in the near future.
